# The S100A10 Subunit of the Annexin A2 Heterotetramer Facilitates L2-Mediated Human Papillomavirus Infection

**DOI:** 10.1371/journal.pone.0043519

**Published:** 2012-08-22

**Authors:** Andrew W. Woodham, Diane M. Da Silva, Joseph G. Skeate, Adam B. Raff, Mark R. Ambroso, Heike E. Brand, J. Mario Isas, Ralf Langen, W. Martin Kast

**Affiliations:** 1 Departments of Molecular Microbiology & Immunology, University of Southern California, Los Angeles, California, United States of America; 2 Department of Obstetrics & Gynecology, University of Southern California, Los Angeles, California, United States of America; 3 Norris Comprehensive Cancer Center, University of Southern California, Los Angeles, California, United States of America; 4 Department of Biochemistry & Molecular Biology, University of Southern California, Los Angeles, California, United States of America; National Institute of Health - National Cancer Institute, United States of America

## Abstract

Mucosotropic, high-risk human papillomaviruses (HPV) are sexually transmitted viruses that are causally associated with the development of cervical cancer. The most common high-risk genotype, HPV16, is an obligatory intracellular virus that must gain entry into host epithelial cells and deliver its double stranded DNA to the nucleus. HPV capsid proteins play a vital role in these steps. Despite the critical nature of these capsid protein-host cell interactions, the precise cellular components necessary for HPV16 infection of epithelial cells remains unknown. Several neutralizing epitopes have been identified for the HPV16 L2 minor capsid protein that can inhibit infection after initial attachment of the virus to the cell surface, which suggests an L2-specific secondary receptor or cofactor is required for infection, but so far no specific L2-receptor has been identified. Here, we demonstrate that the annexin A2 heterotetramer (A2t) contributes to HPV16 infection and co-immunoprecipitates with HPV16 particles on the surface of epithelial cells in an L2-dependent manner. Inhibiting A2t with an endogenous annexin A2 ligand, secretory leukocyte protease inhibitor (SLPI), or with an annexin A2 antibody significantly reduces HPV16 infection. With electron paramagnetic resonance, we demonstrate that a previously identified neutralizing epitope of L2 (aa 108–120) specifically interacts with the S100A10 subunit of A2t. Additionally, mutation of this L2 region significantly reduces binding to A2t and HPV16 pseudovirus infection. Furthermore, downregulation of A2t with shRNA significantly decreases capsid internalization and infection by HPV16. Taken together, these findings indicate that A2t contributes to HPV16 internalization and infection of epithelial cells and this interaction is dependent on the presence of the L2 minor capsid protein.

## Introduction

Human papillomaviruses (HPV) are one of the most common sexually transmitted viruses, and persistent high-risk HPV infections are causally associated with the development of cervical cancers, which are responsible for the deaths of approximately a quarter of a million women each year worldwide [1], [2]. Of the 15 different cancer-causing high-risk HPV genotypes, HPV16 is the most common, leading to approximately 50% of all cervical cancers [3]. Despite these statistics and rigorous efforts in understanding the first steps in HPV16 infection, the entire mechanism of how HPV16 enters and infects human cells is yet to be defined. HPV16 is an obligatory intracellular virus that must gain entry and deliver its circular double stranded DNA to the nucleus of basal epithelium host cells for viral replication, and the capsid proteins play vital roles in these steps [4], [5], [6].

The timing and expression of HPV16 viral genes along with the production of infectious virions is contingent on the differentiation of basal epithelial cells into mature keratinocytes [7]. This contingency has led the majority of the field interested in papillomavirus receptors to use pseudovirions (PsV) and/or virus-like particles (VLP) to study specific aspects of viral internalization and infection. When expressed alone *in vitro*, 360 copies of the major capsid protein L1 can self-assemble into L1 VLP, and when expressed concurrently with the minor capsid protein L2, between 12 and 72 L2 proteins are incorporated per capsid [8], [9]. Although L1 is sufficient to form a VLP, L2 has essential functions for the HPV16 life cycle including DNA incorporation into the viral capsid [10]. HPV16 L1L2 PsV can therefore incorporate DNA within the capsid, making them a useful tool for studying pseudoinfection with reporter genes. To date, it has been demonstrated that HPV infection of epithelial cells is initiated upon viral capsid binding to multiple cell surface receptors, most notably through an initial interaction between L1 and heparan sulfate proteoglycans (HSPG) [11] as well as numerous other potential cell surface receptors such as α_6_β_1/4_ integrin, cyclophilin B, growth factors and growth factor receptors (GF and GFR), and various tetraspanins [4], [12], [13], [14]. However, the entry of HPV16 into cells has been shown to be clathrin-, caveolin-, cholesterol-, and dynamin-independent implying a non-canonical and possibly novel ligand-induced internalization pathway related to macropinocytosis [15], [16].

Though the interaction between L1 and HSPG appears to be the primary initiator of epithelial cell infection, the electrostatic interaction to the negatively charged polysaccharides is generally thought to be non-specific [17], and may lead to a cascade of subsequent capsid conformational changes. The capsid changes that result include furin-mediated proteolytic cleavage of the L2 protein and isomerization by cyclophilins, resulting in decreased affinity of the capsid for primary receptors and increased exposure of the L2 N-terminus to suggested secondary cell surface receptors sites [12], [18], [19]. Additionally, it has been shown that initial binding to HSPG does not mediate HPV uptake and infection, and possible L1 or L2-specific secondary receptors or co-receptors may be involved in the infectious internalization of HPV into host cells [20], [21]. Antibodies against the N-terminus of HPV16 L2 have consistently been shown to inhibit HPV16 infection and several neutralizing epitopes have been described, which may indicate the presence of an L2-specific receptor [22], [23], [24], [25], [26]. Antibodies against L2 amino acids 17–36 are highly conserved and cross-neutralizing against several high risk papillomavirus genotypes [26]. Additionally, antibodies against amino acids 108–126 and 107–122 effectively neutralize HPV16 infection [24], [27]. One of these previously established HPV16 N-terminal L2 neutralizing epitopes has been shown to facilitate HPV16 binding to epithelial cells through an interaction between the L2 protein (aa 108–126) and an unknown cell surface receptor [28], [29]. This region was demonstrated to bind to the surface of the human cervical cancer cell lines HeLa, SiHa, and CaSki, and was additionally shown to facilitate infection on COS-1 cells. Pre-incubation of COS-1 cells with an L2_108–120_ peptide reduced infection by approximately 60% compared to control peptides. Additionally, specific substitutions at aa 108–111 abolished GFP-L2_108–126_ fusion peptide binding to HeLa cells and also reduced PsV infectivity of COS-1 cells [28]. Understanding HPV16-epithelial cell interaction and identifying HPV16 L1- and L2-specific receptors and cofactors involved in internalization and infection on host cells is critical to delineating the events that occur during an active HPV16 infection *in vivo*. However, until now, a specific L2 secondary receptor for HPV16 has not been identified.

A particular local immune event that has been well studied in the context of HPV infection is mucosal co-infection with herpes simplex virus (HSV). Historically, in the 1960s and 1970s, HSV-2 infection was thought to be a possible causative agent for cervical cancer [30], [31]. However, the role of HSV-2 in cervical cancer began to be questioned when HSV-2 DNA was not consistently found in cervical cancer tissues [32]. Subsequently, HPV DNA was detected in the overwhelming majority of cervical cancer tissue and determined to be the causative agent. Nonetheless, the link between HSV and HPV persisted. Recently, it was demonstrated that exposure of human cervical epithelial cells to HSV results in a reduction in the expression of secretory leukocyte protease inhibitor (SLPI), a mediator of mucosal immunity that has been shown to inhibit HSV infection as well as infection by HIV [33]. Mechanistically, SLPI has been shown to inhibit HIV-1 infection of macrophages by binding to and blocking cell surface annexin A2 [34]. Annexin A2 is found at the cell surface as the annexin A2 heterotetramer (A2t) consisting of two annexin A2 monomers and an S100A10 dimer [35], [36], which are co-expressed by basal epithelial cells [37]. Interestingly, the previously mentioned L2_108–126_ peptide was shown to bind significantly less to the A2t-deficient human HepG2 cell line as compared to cervical cancer cell lines that express A2t [28]. Therefore, due to the historical relationship between HSV and cervical cancer, the down regulation of an inhibitory ligand of annexin A2 by HSV, and annexin A2’s implication in different viral entry pathways such as HIV, we hypothesized that the infection of HPV16 is also mediated through A2t. To explore this possibility, we examined the role of A2t in HPV16 infection of epithelial cells as well as the biochemical interactions between A2t and HPV16 capsid proteins. In the process we uncovered a novel role for A2t as an HPV16 L2 specific receptor on epithelial cells and that the specific site of L2 interaction is on the S100A10 subunit of A2t.

## Results

### HPV16 PsV Infection Decreases Following SLPI Treatment or Anti-annexin A2 Antibody Inhibition of A2t

**Figure 1:**
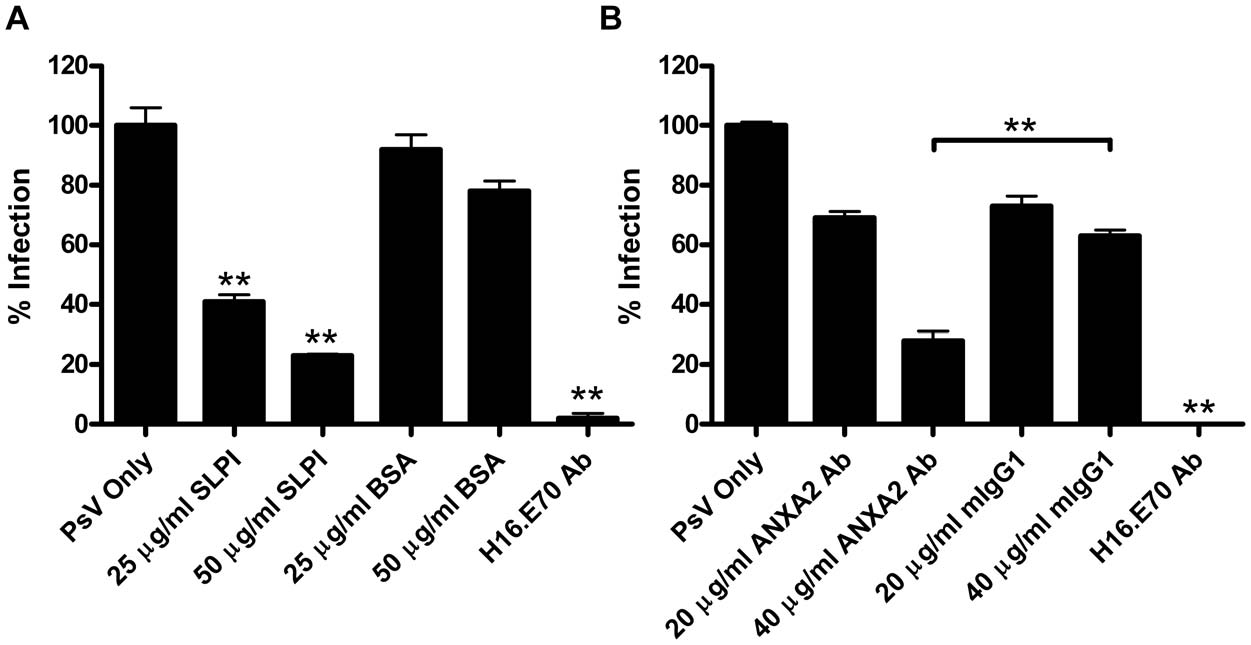
HPV16 PsV infection decreases following SLPI treatment or anti-annexin A2 antibody inhibition of A2t. HaCaT cells were infected with HPV16 pseudovirions containing a GFP plasmid. Infectivity was scored at 48 h post infection by enumerating GFP-positive cells by flow cytometry. (A) Cells were preincubated with increasing amounts of SLPI or BSA for one hour at 4° prior to PsV infection. The mean percentage of HPV16 PsV infected cells (GFP-positive) normalized to the PsV only group ± SD are presented. (**P<0.01 as determined by a two-tailed, unpaired t-test, as compared to the PsV only group). (B) Cells were incubated with increasing amounts of an anti-annexin A2 Ab or isotype control (mouse IgG1) for one hour prior to PsV infection The mean percentage of HPV16 PsV infected cells (GFP-positive) normalized to the PsV only group ± SD are presented (**P<0.01 as determined by a two-tailed, unpaired t-test, as compared to PsV only except where otherwise noted). Each graph is representative of at least two independent experiments.

It was previously demonstrated that SLPI inhibits the infection of HIV-1 through extracellular annexin A2 [34], and HSV causes a sustained down-regulation of SLPI [33]. Therefore, due to the high prevalence of coinfection of HPV and HSV, we predicted that SLPI would inhibit HPV16 internalization if it also utilizes a similar pathway. Additionally, we hypothesized that similar inhibition would be seen with an anti-annexin A2 antibody if SLPI inhibition of HPV16 occurs through A2t. To test this hypothesis, the effect of SLPI and antibody inhibition of A2t on HPV16 infection of epithelial cells was examined *via* HPV16 pseudoinfection of HaCaT cells where reporter gene transduction was used as a measure of HPV16 infectivity. HaCaT cells were incubated with increasing amounts of SLPI or BSA as a control in serum free conditions, and subsequently exposed to PsV containing an expression vector coding for Green Fluorescence Protein (GFP). A significant decrease in pseudo-infection was observed using 25 µg/mL of SLPI, and pseudo-infection further decreased with 50 µg/mL of SLPI compared to negative and BSA controls (approximately 60–80% decrease in pseudo-infection with 25–50 µg/mL SLPI compared to untreated HaCaT cells) (Figure 1A). Similar results were seen on HeLa cells only when PsV infections were done in the absence of FBS, but the presence of FBS during SLPI incubation and PsV infection eliminated the blocking effect of SLPI completely (data not shown), confirming the data of others [38]. It is possible that unidentified FBS proteins either act as competitive substrates of SLPI or block binding of SLPI to A2t.

Next, HaCaT cells were incubated with increasing concentrations (20–40 µg/mL) of an anti-annexin A2 antibody or isotype control before exposure to GFP-vector containing HPV16 PsV. Pseudo-infection of HaCaT cells was significantly reduced at the concentrations of anti-annexin A2 Ab tested compared to PsV only, though some reduction in pseudo-infection was also observed in the isotype control groups (Figure 1B). However, when the 40 µg/mL anti-annexin A2 group is compared to the 40 µg/mL isotype control group, the infectivity in the anti-annexin A2 group was significantly decreased compared to the isotype control. Similar effects of annexin A2 antibody blocking were observed on HeLa cells, verifying our results in another HPV-permissive epithelial cell line (data not shown). The maximum reduction in infectivity due to antibody inhibition of annexin A2 appeared to mirror the maximum decrease due to SLPI inhibition, suggesting the blocking effect of SLPI was due to its affinity for A2t.

### Epithelial Cells Express A2t on the Extracellular Membrane

**Figure 2:**
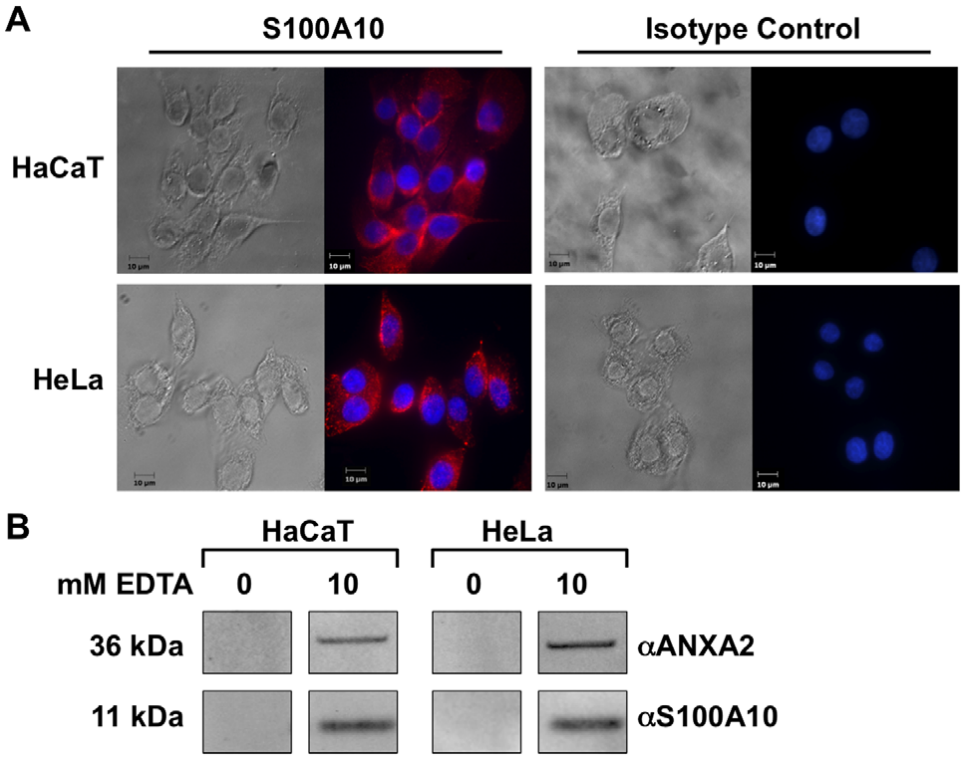
Surface expression of A2t on HaCaT and HeLa human epithelial cell lines. ( A) HaCaT and HeLa cells were incubated with an anti- S100A10 antibody, then incubated with fluorophore-conjugated secondary antibodies, and mounted with DAPI containing media. For control staining, cells were either stained with a mouse or rabbit IgG isotype control followed by secondary antibody staining. Images were acquired using an upright confocal fluorescent microscope. (B) HeLa and HaCaT cells were incubated with PBS supplemented with Ca^2+^ or PBS with increasing concentration of EDTA for 45 min. The supernatants were collected and the presence of ANXA2 and S100A10 were analyzed via Western blot.

A2t is a calcium-binding protein, which can be found on the inner leaflet of the plasma membrane but can translocate to the outer leaflet under certain conditions [39]. To determine whether A2t is found on the extracellular membrane of HaCaT and HeLa cell lines, we visualized the extracellular A2t complex with immunofluorescence microscopy on non-permeabilized cells. The presence of S100A10 was detected as diffuse staining on the surface of HaCaT cells and more punctate staining on the surface of HeLa cells (Figure 2A). Since S100A10 binding to the cell surface is mediated through heterotetramer complex formation with its phospholipid binding partner annexin A2 (reviewed in [36]), positive staining for S100A10 indicates that A2t is found at the cell surface. To further demonstrate A2t cell surface localization, cells were incubated with the Ca^2+^ chelating agent ethylenediaminetetraacetic acid (EDTA) for 45 min, which releases extracellular A2t from membranes. The supernatants were collected and the presence of A2t was analyzed via Western blot. In HaCaT and HeLa cells, both extracellular A2t components, annexin A2 and S100A10, were detected in the supernatants of EDTA-treated cells (Figure 2B). Collectively, these data indicate that extracellular A2t is found in abundance on the cell surface of both epithelial cell types.

### HPV16 Binds to A2t on the Cell Surface of HeLa Cells

**Figure 3:**
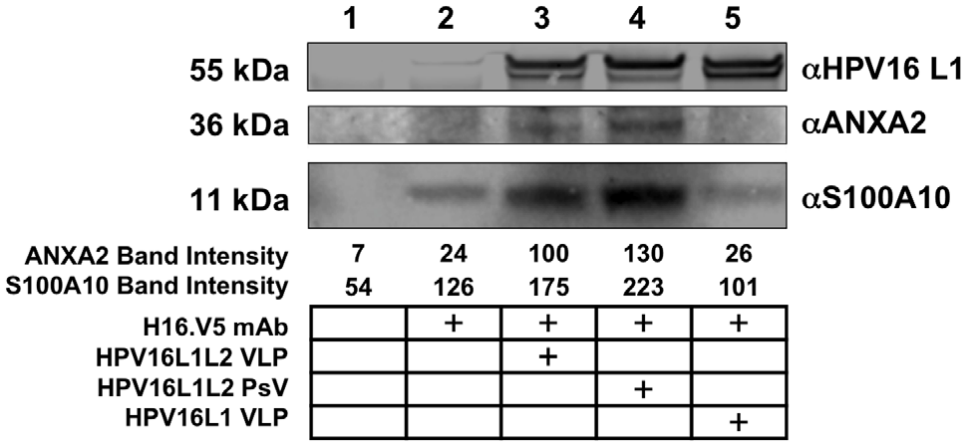
HPV16 binds to A2t on the cell surface of HeLa cells. HeLa cells were incubated with HPV16 L1L2 VLP, HPV16 PsV or HPV16 L1 VLP for 1 hour at 37°C. The cells were washed and surface proteins cross-linked. HPV16 VLP and PsV were precipitated out of cell lysates with an anti-L1 antibody (H16.V5) conjugated to magnetic beads. Elutions were analyzed via Western blot for the presence of ANXA2 and S100A10. Band density was determined by Licor Odyssey imaging software. HPV L1 western blot shows equivalent amounts of HPV16 particles were precipitated in lanes 3–5. The table below the figure indicates which components were included in each treatment. Data are representative of at least three independent experiments.

After epithelial cell surface expression of A2t was demonstrated, we then tested whether HPV16 interacts with A2t at the cell surface through extracellular co-immunoprecipitation (Co-IP). HeLa cells were incubated with HPV16 PsV, HPV16 L1L2 VLP, or HPV16 L1 VLP followed by incubation with an extracellular cross-linking agent. The cells were then lysed and HPV16 was precipitated out of solution with an anti-L1 conformational antibody, and the co-immunoprecipitation of HPV16 particles and A2t was visualized by Western blot (Figure 3). Western blot images and band density quantitation show that both A2t components, annexin A2 and S100A10, were greatly enriched in the presence of HPV16L1L2 VLP (Figure 3, Lane 3) compared to HPV16 L1 VLP (Figure 3, Lane 5) or negative controls (Figure 3, Lanes 1–2). DNA-induced virus particle structural changes have been previously reported, and minor differences between VLP and PsV internalization have been observed [40], [41]. However, here we show that both HPV16 L1L2 VLP and L2-containing HPV16 PsV (Figure 3, Lane 4) showed similar increased interactions with A2t, indicating that any minor structural changes induced by incorporation of DNA in PsV does not affect accessibility of the exposed L2 portion on the particle surface. These findings suggest that HPV16 is binding to A2t or an A2t-containing complex at the cell surface of epithelial cells and this interaction is dependent on the presence of L2 since A2t did not enrich with L1 VLP.

### HPV16 L2_108–126_ is Exposed on L1L2 VLP and PsV

**Figure 4:**
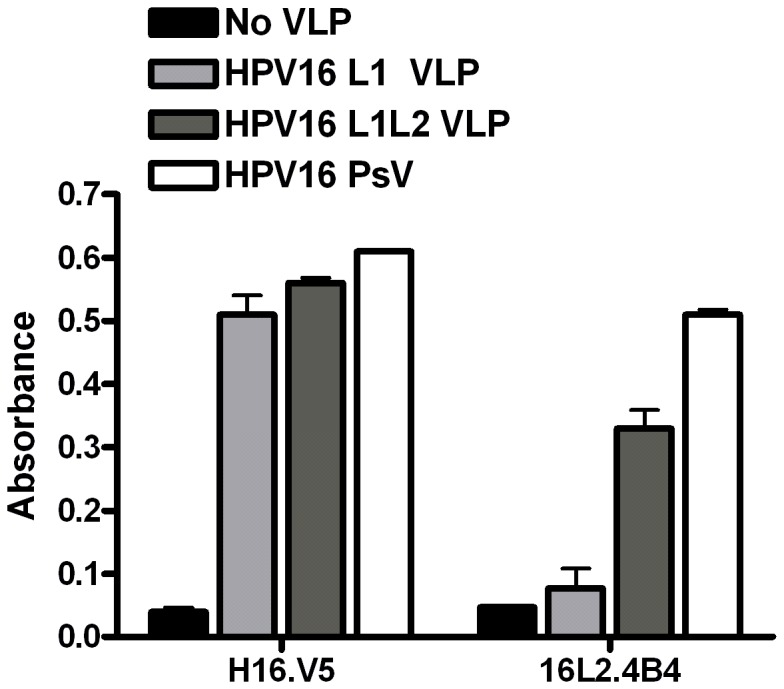
HPV16 L2_108–126_ is exposed on HPV16 L1L2 VLP and HPV16 PsV. ELISA plate wells were coated with 500 ng of HPV16 L1 VLP, HPV16 L1L2 VLP or HPV16 PsV and subsequently incubated with anti-L1 H16.V5 or anti L2_108–126_ 16L2.4B4 antibodies. Secondary HRP-conjugated secondary antibodies were added prior to the substrate. In control experiments, no VLP were used. The graph represents the mean absorbance measured at 490 nm ± SD of triplicate wells. Data was repeated in three independent experiments.

It has been demonstrated that a previously described neutralizing epitope of the minor capsid protein (L2_108–126_) can bind to the surface of several human cell lines including HeLa, SiHa, and CaSki, all of human cervical cancer origin [23], [28]. This same region of HPV16 L2 has also been shown to be involved in infection of COS-1 cells [28], and homologous regions of L2 from other papillomaviruses (PV) have been suggested to be exposed on PV particles that are required for infection [29]. Therefore we investigated if L2_108–126_ was exposed on HPV16 VLP and PsV with an enzyme-linked immunosorbent assay (ELISA). ELISA plate wells were coated with HPV16 L1 VLP, HPV16 L1L2 VLP, or HPV16 PsV and subsequently stained with an antibody specific to L2_108–120_ (clone 16L2.4B4) which has previously been shown to neutralize HPV virus [27]). Similarly, it has been shown that polyclonal antibodies against aa 107–122 effectively neutralize HPV16 infection [24], [25]. We observed antibody binding to HPV16 L1L2 VLP and HPV16 PsV, and minimal antibody binding observed with L1 VLP, suggesting that this particular region of L2 is exposed on mature HPV16 particles (Figure 4). The observed absorbance was greater with PsV, which could be attributed to an increased number of L2 proteins incorporated into the PsV capsid, as it has been shown that PsV can be produced with up to 72 molecules of L2, whereas 12 L2 molecules are generally associated with L1L2 VLP [9], [42]. All particles were equally detected with the conformation-dependent and neutralizing L1 antibody, H16.V5, indicating that the capsid structures were intact.

### HPV16 L2_108–126_ Binds Specifically to the S100A10 Subunit of A2t

Next, we wanted to determine if there was a specific interaction between the L2_108–126_ peptide and A2t using electron paramagnetic resonance (EPR) spectroscopy. EPR is a method of magnetic resonance spectroscopy measuring unpaired electrons whose underlying basic concepts are not unlike the more widely used technique of nuclear magnetic resonance (NMR) (reviewed in [43]). When coupled with site-directed paramagnetic-labeling, such as the attachment of a moiety with an unpaired electron to a cysteine residue, EPR can be used to obtain structural information within a small vicinity of the paramagnetic-label’s positions along the protein sequence. As a paramagnetic-label becomes locally constrained, such as when bound to another protein, its spectra will drastically change as visualized by a decrease in line amplitude and increased line-broadening. Therefore, EPR provides an *in vitro* biochemical method that measures protein-protein interactions by monitoring changes in the spectra recorded for a labeled protein in different experimental settings.

**Figure 5:**
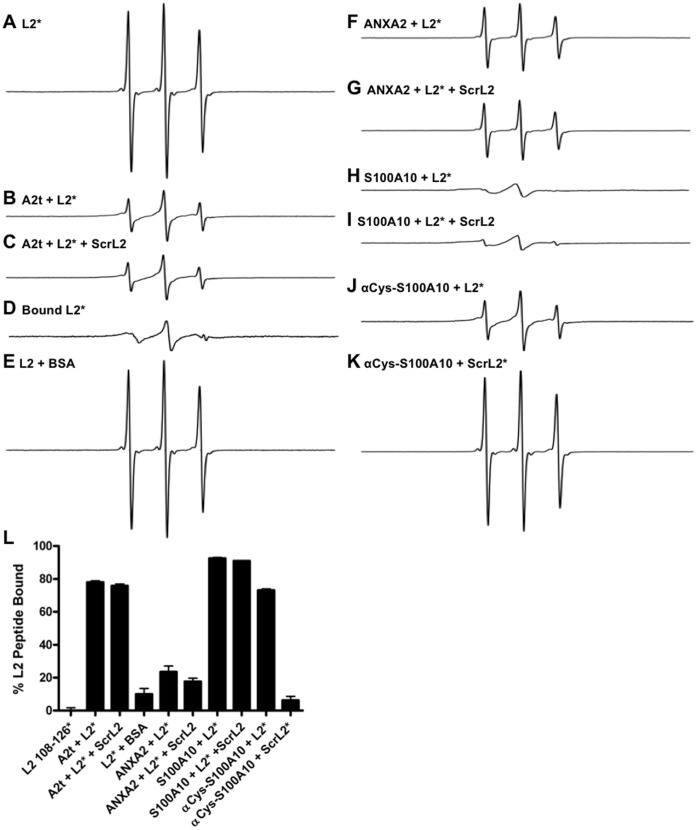
HPV16 L2_108–126_ peptide binds to the S100A10 subunit of A2t protein with sequence specificity. HPV16 L2 peptide was paramagnetic-labeled (*denotes the attachment of the paramagnetic-label) and analyzed for binding to purified A2t, S100A10 and ANXA2 protein in vitro by electron paramagnetic resonance. (A) The first derivative spectrum of L2_108–126_ peptide free from ligands in solution. (B) The first derivative spectrum of L2_108–126_ combined with A2t. (C) The first derivative spectrum of L2_108–126_ combined with A2t in the presence of the control peptide (5∶1). (D) The result of subtraction of spectrum A from spectrum B representing the bound spectrum of the labeled peptide. (E) The first derivative spectrum of L2_108–126_ combined with BSA. (F) The first derivative spectrum of L2_108–126_ combined with ANXA2. (G) The first derivative spectrum of L2_108–126_ combined with ANXA2 in the presence of the scrambled peptide (5∶1). (H) The first derivative spectrum of L2_108–126_ combined with S100A10. (I) The first derivative spectrum of L2_108–126_ combined with S100A10 in the presence of the scrambled peptide (5∶1). (J) The first derivative spectrum of L2_108–126_ combined with cysteine-blocked S100A10 (αCys-S100A10). (K) The first derivative spectrum of the paramagnetic-labeled scrambled peptide combined with cysteine-blocked S100A10. (L) Quantification of relative % bound of all treatments as measured through spectra subtraction and double integration expressed as the mean of three separate experiments ± SD.

For our purposes, an N-terminal cysteine (with a 3 native aa spacer) contained in the L2_108–126_ peptide (C-IVS-LVEETSFIDAGAPTSVPSI) was paramagnetic-labeled (an unpaired-electron-containing small molecule was cross-linked via a disulfide bond to the peptide), and its spectra were analyzed after incubation with and without purified human recombinant A2t or controls. The spectrum of the paramagnetic-labeled L2_108–126_ peptide alone displayed sharp, high amplitude lines indicative of a non-constrained paramagnetic label (Figure 5A), whereas the spectrum of L2_108–126_ incubated with A2t resulted in characteristic line-broadening and a decrease in amplitude (Figure 5B), which is an indication of a constrained paramagnetic label suggesting strong protein-protein binding (78.0% of L2_108–126_ bound to A2t). To examine sequence specificity of the L2_108–126_ peptide binding to A2t, a scrambled version of the L2_108–126_ peptide (ScrL2) labeled with a non-paramagnetic and chemically similar analog (C-IVS-IESPVSDTALGTPEIFVSA) was examined for its ability to compete for the interaction between A2t and the wild type-paramagnetic-labeled L2_108–126_ (WT L2_108–126_) peptide using a 5∶1 molar ratio of ScrL2 to WT L2_108–126_. No significant changes in spectra signal were observed, suggesting that the interaction is sequence specific (75.8% WT L2_108–126_ bound to A2t with ScrL2 at 5∶1 molar ratio) (Figure 5C). Relative percent binding was calculated by measuring the amount of bound and unbound labeled peptide in each experiment through subtraction of the bound and unbound spectra (see materials and methods for details) (spectra Fig. 5D, quantified Fig. 5L), and this data was used to calculate a relative binding constant (K) of 10^5^ M^−1^ for the L2 peptide and A2t using the formula K = [AB]/[A][B] where [AB] is the concentration of L2_108–126_ bound to A2t based on the percent bound and the initial concentration (i.e. 20 mM × 78.0%), and [A] and [B] are the concentrations of unbound L2_108–126_ and A2t respectively. These results demonstrate that a specific interaction exists between HPV16 L2_108–126_ and A2t. As a negative control to test for protein binding specificity, the L2_108–126_ peptide was mixed with Bovine Serum Albumin (BSA). The EPR spectra displayed sharp, non-broadened lines with high-amplitudes indicating that L2_108–126_ does not bind BSA in a significant manner (Figure 5E).

Similar EPR assays were then performed with the annexin A2 and S100A10 subunits of A2t to determine the site of interaction between L2_108–126_ and A2t. When the L2 peptide was mixed with annexin A2 alone, only 23.6% peptide binding was observed (spectra Figure 5F and quantified in Figure 5L). This interaction was partially competed off in the presence of the paramagnetic-analogue labeled ScrL2 peptide (17.7% L2_108–126_ binding) (spectra Figure 5G and quantified in Figure 5L). When the L2 peptide was mixed with S100A10, there was an observed binding of 92.5% (Figure 5H) that was minimally competed off in the presence of the ScrL2 peptide (91.0% L2_108–126_ binding) (spectra Figure 5I and quantified in Figure 5L). This data was used to calculate a relative binding constant (K) of 1.5×10^5^ M^−1^ between L2_108–126_ and S100A10. As an additional control, the paramagnetic-labeled ScrL2 peptide was analyzed for direct interaction with S100A10 to further test for sequence specificity. S100A10 was treated with a cysteine blocking agent to prevent non-specific disulfide bond interactions, followed by incubation with the paramagnetic-labeled WT L2_108–126_ or paramagnetic-labeled ScrL2. Under these conditions, a significant interaction with the WT L2_108–126_ was observed but the ScrL2 peptide did not show any significant direct binding to S100A10 (73.1% compared to 6.3%, respectively) (spectra Figure 5J–K and quantified in Figure 5L). The cysteine block prevents non-specific interactions due to the presence of surface cysteines, and was found to denature annexin A2 making it unfeasible to test direct ScrL2 binding to annexin A2 or A2t. Taken together these results indicate that L2_108–126_ specifically interacts with the S100A10 subunit of A2t with sequence specificity.

### Specific Mutations in HPV16 L2_108–111_ Decrease HPV16 Binding to A2t and HPV16 PsV Infectivity

**Figure 6:**
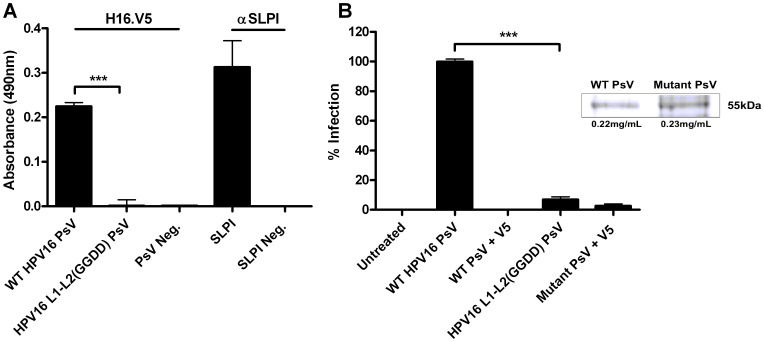
Mutations in HPV16 L2_108–111_ reduce PsV binding to A2t and PsV infectivity. (A) ELISA plate wells were coated with 500 ng of A2t prior to overnight incubation with 400 ng HPV16 PsV or HPV16 L1–L2(GGDD) mutant PsV and subsequently incubated with mouse anti-L1 H16.V5 or goat anti-SLPI antibodies. Anti-mouse and anti-goat HRP-conjugated secondary antibodies were added prior to the substrate. In control experiments, no ligands were used. The graph represents the mean absorbance measured at 490 nm ± SD (***P<0.001 as determined by a two-tailed, unpaired t-test between WT and mutant PsV). (B) HaCaT cells were infected with wild type (WT) or mutant (L2_108–111_ LVEE to GGDD) HPV16 pseudovirions containing a GFP plasmid. Infectivity was scored at 48 h post infection by enumerating GFP-positive cells by flow cytometry. The mean percentage of HPV16 PsV infected cells (GFP-positive) normalized to the WT PsV group ± SD are presented of two combined independent experiments. Inset shows the L1 band of a coomassie blue stained SDS-PAGE gel loaded with an equivalent amount of WT and mutant PsV used in the infectivity assays. (***P<0.001 as determined by a two-tailed, unpaired t-test between WT and mutant PsV group).

It has been previously reported that substitutions in aa 108–111 (LVEE to GGDD) of HPV16 L2 decrease the binding of the L2_108–126_ peptide binding to HeLa cells and reduce infectivity of HPV16 pseudovirus on COS-1 cells [28]. Therefore to test whether this region is involved in A2t binding, we generated HPV16 PsV incorporating these mutations in the L2 binding site and investigated if these substitutions affected the interaction between HPV16 and A2t using an ELISA assay (Figure 6A). ELISA plate wells were either coated with A2t or left untreated before the wells were blocked with casein and subsequently exposed to HPV16 PsV or mutant HPV16 L1–L2(GGDD) PsV. We found that the binding of the mutant PsV to A2t was significantly reduced compared to the non-mutated WT PsV, and that the measured absorbance was not significantly different than negative controls that were incubated in the absence of HPV16 particles. As a positive control, A2t coated wells were incubated with SLPI and significant binding was observed. Thus by mutational analysis, our data suggest that in an intact virus particle, the region L2_108–111_ is involved in direct A2t binding.

Next, the effect of the mutations on HPV16 infectivity was examined. HaCaT cells were either treated with an equal amount of PsV infectious units of GFP-plasmid containing wild-type HPV16 PsV or mutant HPV16 L1–L2(GGDD) PsV. The mutation in L2 caused a significant (>10-fold) reduction in the percent of GFP-positive HaCaT cells compared to the group treated with the wild-type PsV (Figure 6B). To ensure that the WT PsV and mutant PsV did not differ in their capsid:infectious unit ratios, a Coomassie Blue stain was used to quantify L1 in each PsV preparation and showed that equivalent numbers of particles were used in the wild-type and mutant groups (Figure 6B). Immunoblot analysis confirmed that mutation of L2 did not affect incorporation of the L2 protein into the pseudovirion compared to WT PsV (Figure S1). Furthermore, to test that the L2 mutation did not adversely affect encapsidation of the reporter plasmid, the number of reporter plasmid copies/ng of capsid proteins was quantified by qPCR and showed that the mutation did not decrease DNA packaging (3.3×10^6^ copies/ng of the WT PsV and 5.6×10^6^ copies/ng of the mutant PsV). Together with the EPR data, this suggests that there is a specific interaction between L2_108–126_ and the S100A10 subunit of A2t that is associated with HPV16 infectivity.

### shRNA Knockdown of A2t Reduces Internalization of HPV16 L1L2 VLP and Infectivity of HPV16 PsV

**Figure 7:**
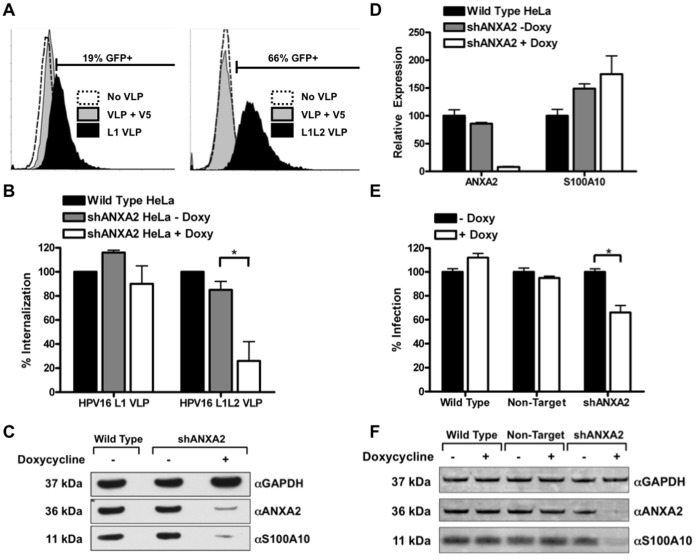
shRNA knockdown of A2t reduces internalization of HPV16 L1L2 VLP and infectivity of HPV16 PsV. (A) HPV16 L1 VLP were fluorescently labeled with CFDA-SE, and the percent of infected cells was measured as the percent that were CFDA-SE positive after exposure to VLP for 1 hour as measured by FACS. To control for free label false positives, cells were treated with VLP pre-incubated with a neutralizing anti-L1 antibody (H16.V5). An identical experiment was performed using HPV16 L1L2 VLP. Both histograms are representative examples of two experiments done in triplicate. (B) HeLa cells were left untreated or transduced with a doxycycline inducible pTRIPZ Tet-On lentiviral vector containing an shRNA against ANXA2. Single cell clones treated with or without doxycycline were incubated with labeled HPV16 VLP for 3 hours at 37° and assessed by FACS. The mean percentage of uptake normalized to the wild type group ± SEM of three independent experiments is presented. (C) Protein was collected from cell populations used in the internalization assay for analysis of ANXA2 and S100A10 via Western blot. GAPDH served as a loading control. (D) mRNA was collected from cell populations in the uptake assay for quantitative RT-PCR analysis of ANXA2 and S100A10 expression. The mRNA expression levels were normalized to GAPDH and the graph is a representative example of an experiment performed in triplicate ± SD. (E) Wildtype HeLa cells or HeLa cells stably transduced with a doxycycline inducible lentiviral vector containing shRNA against ANXA2 or control non-target lentiviral vector were infected with GFP plasmid containing HPV16 pseudovirus. Infection was scored 48 h later by enumeration of GFP-positive cells by flow cytometry. The mean percentage of HPV16 PsV infected cells (GFP-positive) normalized to the no doxycycline treated groups ± SD are presented. (*P<0.05 and **P<0.01 as determined by a two-tailed, unpaired t-test, as compared to the no doxycycline-treated groups). Figure is representative of two independent experiments. (F) Protein was collected from cell populations used in the infection assay for analysis of ANXA2 and S100A10 via Western blot. GAPDH served as a loading control.

To investigate the contribution of L2 to HPV16 capsid internalization, an internalization assay was performed with carboxyfluorescein diacetate, succinimidyl ester (CFDA-SE) labeled L1 and L1L2 VLP (Figure 7A) measured *via* FACS on HeLa cells. Fluorescence of CFDA-SE occurs when acetate groups are cleaved by intracellular esterases, therefore only VLP that have been internalized are detected. The percent of CFDA-SE positive cells was significantly greater after exposure to L1L2 VLP (66% CFDA-SE positive after 1 hour) compared to L1 VLP (19% CFDA-SE positive after 1 hour), which shows that L2 increases the efficiency and/or rate at which the particles enter these cells. These findings suggest that there may be alternate pathways associated with the L2 protein, and coincide with findings previously reported for HPV type 31 [44].

Next we wanted to determine the effect of down-regulating A2t on both L1 and L1L2 VLP internalization. It has been previously demonstrated that A2t light chain S100A10 is post-transcriptionally modified by annexin A2, and protein knockdown of annexin A2 leads to degradation of S100A10 [45], [46]. Therefore, in order to down regulate A2t expression in epithelial cells, we transduced HeLa cells with a lentiviral vector encoding a short hairpin (sh) RNA against annexin A2 mRNA (shANXA2) whose expression was doxycycline inducible. HPV16 L1 VLP and L1L2 VLP uptake was tested in transduced cells that were either treated with doxycycline or left untreated. We found that shRNA knockdown of A2t reduced internalization of L1L2 VLP by 75% compared to wild type cells in the doxycycline treated transduced group compared to only 15% in the non-treated control group (p<0.05; Students t-test comparing doxycycline to non-doxycycline control) (Figure 7B). L1 VLP internalization was not decreased in doxycycline treated transduced cells. Knockdown of annexin A2 and S100A10 protein levels was confirmed by Western blot where we found significant reductions of both proteins in the doxycycline-treated group (Figure 7C). We also confirmed previous findings by quantitative RT-PCR that downregulation of ANXA2 mRNA does not negatively affect S100A10 mRNA levels (Figure 7D) [45]. These results show that shRNA against ANXA2 is sufficient and effective in reducing both components of A2t at the protein level while reducing the potential off-target effects of using shRNA against both targets. These data also indicate that knockdown of A2t does not affect general endocytosis or intracellular trafficking because L1 VLP internalization was unaffected. Together with the preliminary internalization (Figure 7A) these data support the hypothesis that A2t facilitates L2-mediated HPV16 internalization.

Lastly, we tested the effects of stable shRNA mediated knockdown of A2t on the pseudo-infection of epithelial cells. HeLa cells were transduced with a lentiviral vector coding for an shRNA against ANXA2 as previously mentioned, or a non-silencing analog. The cells were then either treated with doxycycline or left untreated before exposure to GFP plasmid containing HPV16 PsV. A significant reduction in pseudo-infection was observed in the anti-ANXA2 shRNA group that was treated with doxycycline compared to its non-doxycycline control group (Figure 7E). The wild type and non-silencing transduced HeLa cells showed no decrease in HPV16 pseudo-infection when treated with doxycycline. A Western blot confirmed that the doxycycline-treated shANXA2 group showed a marked decrease in A2t compared to all other groups (Figure 7F). Taken together, these results indicate that HPV infection is decreased in epithelial cells when A2t is either blocked by external ligands or antibodies directed towards A2t or when the complex is genetically knocked down in HPV16 susceptible cell lines.

## Discussion

The concept of viruses binding to a single receptor and subsequently entering cells through a single uptake mechanism has been challenged [47], [48]. Instead, a more complex picture is forming where specific co-receptors and multiple attachment sites lead eventually to viral entry by one or multiple uptake mechanisms. Numerous binding receptors and cofactors for HPV16 L1 are now recognized, building a complex and cooperative paradigm of HPV16 infection, but the identity of an L2-specific receptor has remained elusive despite the identity of known L2 neutralizing epitopes. The highly conserved HPV16 neutralizing L2_108–126_ epitope has been shown to be associated in the binding of HPV16 to several cervical cancer cell lines, but a cell surface receptor was never identified [28]. To explore the role of A2t as an HPV16 receptor on epithelial cells, both HaCaT and HeLa cells were used, as they are two common human cell lines used in HPV entry and life cycle research. To stay consistent within systems in our study, HaCaT cells were used primarily for infection experiments and results were then verified on HeLa cells. HeLa cells were used for knockdown and Co-IP assays where lentiviral transduction and expansion of high numbers of cells was more efficient. Collectively, our results identify A2t as a novel receptor complex for HPV16 and support the hypothesis that HPV16 L2 binds to the S100A10 subunit of A2t and facilitates HPV16 infection of epithelial cells.

The use of VLP and PsV to study HPV binding, entry, and infectivity has been widely employed [16], [19], [29], [38], [49], [50]. The primary difference between L1 VLP and L1L2 VLP is the presence of the L2 protein, making L1L2 VLP the preferred particle type to study the role of L2 in binding and internalization. In this regard, VLP were utilized to highlight differences in binding and internalization between VLP containing L2 and those that do not in order to establish an association between L2 and A2t. Additionally, our data showed that DNA-containing HPV16 PsV bound similarly to the A2t complex as HPV16 L1L2 VLP, indicating that any DNA-induced structural changes do not impact association with A2t, and that the cell surface-binding motif L2_108–126_ is exposed on both HPV16 L1L2 VLP and PsV. Here, HPV16 PsV containing a reporter plasmid coding for GFP were used to demonstrate that A2t facilitates infection, and mutations in L2 were used to demonstrate a dependence on a specific region of the L2 protein that is exposed on the surface of mature capsids.

Annexin A2 is found in the cytoplasm as a 36 kDa monomer and at the cell surface as a 90 kDa heterotetramer (A2t), which consists of two annexin A2 monomers bridged non-covalently to an S100A10 dimer [35]. S100A10, also known as p11 or annexin A2 light chain, is a member of the S100 family of phospholipid-binding proteins [36]. The S100A10 protein is post-translationally stabilized by annexin A2 [45], [51], and in a majority of cells, A2t is the predominant form [35]. This was confirmed in our own experiments where it was found that shRNA against ANXA2 mRNA was sufficient to down regulate the entire A2t complex, whereas the mRNA of S100A10 was increased, which may have been a cellular response to the knockdown of annexin A2. In normal epithelium, annexin A2 expression is confined to the basal and suprabasal cells and the protein’s cellular location is consistently observed at the cell membrane in these cells [37]. More specifically, an immunohistochemical study investigating the differential expression of annexins found that annexin A2 was expressed on basal cells of the cervix [52]. These observations demonstrate that the expression of A2t mirrors the tropism of HPV16 infection *in vivo*.

Annexin A2 has been proposed to function in exocytosis, endocytosis, cell adhesion, membrane fusion and membrane trafficking [53]. Of particular interest to us, annexin A2 has been shown to play a key role in the binding and uptake of a variety of different viruses. Annexin A2 has been shown to bind directly to cytomegalovirus (CMV) virions [54] and enhance CMV-membrane fusion 54,55. In addition to CMV, it was shown that annexin A2 is a receptor for respiratory syncytial virus (RSV) and that this binding can be inhibited by an antagonist, suggesting a potential for inhibitors of annexin A2 as treatments for RSV infections [56]. Most recently, annexin A2 was shown to bind to the capsid of enterovirus 71, which was associated with an increase in viral infectivity [57]. As previously stated, annexin A2 was shown to be a cofactor for HIV-1 infection in macrophages [34], but until now, annexin A2 has never been associated with HPV16. Our Co-IP data indicate that HPV16 interacts with A2t at the cell surface, fulfilling the definition of a receptor molecule, though we cannot exclude the possibility that additional proteins are present within a larger complex. However, we believe it is unlikely that HPV16 binding to A2t is mediated indirectly by association with other cell surface binding proteins or heparin sulfate, since our EPR data show a direct physical interaction between the L2_108–126_ peptide and A2t, and specifically with the S100A10 subunit of A2t, in the absence of other cellular proteins. Furthermore, our ELISA data indicates that HPV16 PsV can bind directly to A2t in the absence of other cellular components. However, binding of HPV particles to heparin sulfate *in vivo* may increase the overall avidity of A2t binding, since A2t has also been shown to interact with heparin in a Ca^+2^ dependent manner [58].

It has been previously reported by us that HPV16 entry into human Langerhans cells occurs via a non-clathrin, non-caveolin, macropinocytic-related pathway [16]. More recently it has been shown that entry into HaCaT and HeLa epithelial cell lines occurs by a clathrin-, caveolin-, cholesterol-, and dynamin-independent pathway which is suggested to be a novel ligand-induced internalization pathway related to macropinocytosis [15]. Our current study identifies A2t as a novel L2-specific HPV16 receptor that is involved in internalization and infection of epithelial cells, but future studies are needed to determine where A2t may fit in this proposed ligand-induced macropinocytosis related pathway and whether previously identified HPV receptors and cofactors such as HSPG, integrins, growth factors and growth factor receptors, tetraspanins and cyclophilins actively interact with A2t during HPV internalization and infection. There remains a possibility that these molecules work together in a complex that initiates a novel HPV16 endocytic pathway. Other viruses associated with annexin A2 also utilize similar molecules during infection. For instance, CMV uses HSPG for tethering and β1 integrins for fusion and internalization (reviewed in [59]). Moreover, HIV-1 binding to macrophages is associated with HSPG and integrins in addition to annexin A2 before transfer to CD4 and CCR5 [34], [60]. This fits with the emerging concept that viruses use multiple specific receptors and co-receptors that work simultaneously to lead to viral entry, either via a single or multiple pathways, and provides a strong rationale to study how these receptors and pathways either work together or independently. The predominant protein exposed on the capsid surface is the L1 major capsid protein, against which most HPV neutralizing antibodies are generated. While there is some evidence to suggest that furin cleavage of L2 causes a conformational change within L1 that may expose a secondary receptor binding epitope (reviewed in [61]), there is no requirement that this putative secondary receptor binds to the L1 protein. L1 only-containing PV are neither infectious nor occur in nature, but are rather a tool used by scientists as a result of the self-assembling properties of L1 capsomers. Additionally, the N-terminal region of L2 is highly conserved among PV types, and can induce broadly cross-neutralizing antibodies that are capable of preventing capsid binding to the cell surface post HSPG binding and furin cleavage [62], which also suggests that the L2 protein could be a potential receptor binding protein. However our data do not exclude the possibility that additional L1 secondary receptors also exist.

Tetraspanins (CD63/CD151), α_6_β_1/4_ integrins, GF and GFR, and cyclophilin B are all cell surface associated proteins that have been implicated in HPV entry into epithelial cells [4], [12], [14], [50]. It is tempting to speculate that the recruitment of A2t to the cell membrane and subsequent translocation to the cell surface is linked to the binding of HPV16 to integrins and subsequent activation of focal adhesion kinases and src-family kinases and their respective signaling cascades, which notably have been shown to cause translocation of A2t to the cell surface [39], [63], [64], [65], [66]. Consequently, early binding of HPV16 to integrins has the potential to recruit A2t to the specific site of cell membrane interaction. Though currently inferential, these relationships lead to the idea that a signal transduction cascade may be in place where the binding of HPV16 through primary receptors leads to the local recruitment and subsequent translocation of additional A2t to the cell surface where they act as secondary binding and internalization receptors, and gives further reason to investigate the synergy between A2t and other HPV16 receptors. Future studies will aim to elucidate a direct role for A2t in the specific endocytosis of HPV as endocytosis has already been proposed as a function of A2t.

In this study, we used a unique method to determine whether a conserved region of HPV16 L2 binds directly to A2t in vitro, and more specifically to the S100A10 subunit of the heterotetramer. Site-directed paramagnetic-labeling coupled with EPR is a powerful well-established biochemical technique that measures structural changes in proteins by observing energy absorbed by a paramagnetic system in a magnetic field (reviewed in [43]). This technique is particularly useful in studying protein-protein interactions, especially peptide sequences devoid of cysteines and paramagnetic centers such as the N-terminal L2_108–126_ peptide. While this technique has been used to measure other viral protein interactions with ligands and/or protein partners [43], it had not yet been employed for HPV research, and can now provide another tool to study HPV-receptor interactions. Based on our data, the HPV16 L2_108–126_ peptide and A2t interacted strongly with a calculated relative binding constant (K) of 10^5^ M^−1^ in the absence of other proteins. To put this in perspective, a soluble analogue of HSV glycoprotein D has been shown to bind to a soluble analogue of its well-established receptor HVEM within the same order of magnitude (measured with surface plasmon resonance) [67]. Furthermore, our data indicate that this peptide had a minimal interaction with the annexin A2 subunit of A2t, but had a very strong interaction with the S100A10 subunit suggesting that S100A10 is the site of interaction for L2 on A2t. Future EPR analysis and mutational studies will aim to address the exact site where HPV16 binds to the S100A10 portion of A2t.

We demonstrate that SLPI, a ligand of annexin A2, can inhibit HPV16 infection of HaCaT cells. SLPI has shown anti-bacterial and anti-fungal activity aside from its noted anti-protease activity (reviewed in [68]). Human epithelial and myeloid cells constitutively secrete SLPI, and cellular production can be significantly upregulated in response to various stimuli [69]. Concentrations in cervical mucosal fluids are commonly reported at 1 µg/ml [70], but are likely to be more concentrated at the cell surface. In addition, SLPI levels in cervical mucus have been measured midcycle as high as 78 µg/mL [71]. Thus, the concentrations of SLPI used in our blocking studies fall within physiological levels found *in vivo*. Interestingly, SLPI has been reported at its highest concentrations in saliva and upper airway (25–100 µg/mL) [72], [73], which has implications in HPV-related head and neck cancers where SLPI has already been reported to be significantly reduced [74]. A previous report in which the authors screened a selection of antimicrobial peptides and proteins for inhibition of PsV infectivity on HeLa cells showed no reduction in HPV16 infection by SLPI [38]. However, those studies were performed in the presence of FBS, which had the potential to mask any inhibitory effects due to the presence of potential SLPI substrates in the serum. In this regard, we also observed no reduced infection when SLPI was used in the presence of FBS (data not shown).

Infection of human cervical epithelial cells with HSV results in a significant and sustained reduction in SLPI levels [33]. In the context of our results demonstrating that SLPI may play a role in HPV16 infection by inhibiting entry into epithelial cells, the evidence showing that HSV down-regulates SLPI may explain the epidemiological association between HSV and HPV induced cervical cancer. HSV infection may actually increase the likelihood of HPV entry, infection and/or persistence by suppressing a mucosal ligand of the HPV receptor A2t. While further studies need to be conducted to test this hypothesis, this is an exciting and important connection that has the potential to cohesively link decades’ worth of epidemiological data, and show a potential protective role for SLPI against HPV16 infection based on its interaction with A2t, making these findings significant across multiple disciplines.

## Materials and Methods

### Cell Cultures, Antibodies, and Recombinant Proteins

HeLa cells (ATCC, Manassas, VA) are human epithelial cells derived from cervical cancer, and were maintained in complete media (IMDM, 10% FBS, 1X PenStrep) at 37°C with 5% CO_2_ (Lonza, Walkersville, MD). HaCaT cells (Cell Lines Service, Eppelheim, Germany) are *in vitro* spontaneously transformed human keratinocytes derived from histologically normal skin [75] and were maintained in Defined Keratinocyte Serum-Free Media (KSFM) (Invitrogen, Carlsbad, CA) with manufacturer provided KSFM growth supplement (including insulin, EGF, and FGF) at 37°C with 5% CO_2_.

The following antibodies were used in this study: mouse-anti-annexin A2 (BD Biosciences, San Jose, CA); mouse-anti-S100A10 (BD Biosciences); rabbit anti-GAPDH (Cell Signaling, Danvers, MA); H16.V5 mouse-anti-L1 (a gift from Neil Christensen, Penn State); H16.E70 mouse anti-HPV16 L1 (a gift from Neil Christensen); mouse anti HPV16 L1 (Camvir-1; BD Biosciences); 16L2.4B4 mouse-anti-L2_108–120_ (a gift from Neil Christensen); rabbit anti-HPV16 L1 polyclonal antibody (a gift from Martin Müller, DKFZ, Germany); rabbit anti-HPV16 L2 polyclonal antibody (a gift from John Schiller, NIH); rabbit-anti-mouse HRP (Promega, Madison, WI); rabbit anti-goat HRP (Promega); goat anti-human SLPI (R&D Systems, Minneapolis, MN); AlexaFluor 680 goat-anti-rabbit IgG (Invitrogen) and IRDye 800 donkey-anti-mouse IgG (Rockland, Gilbertsville, PA). Recombinant human (rhu)-SLPI was purchased from R&D Systems.

Recombinant annexin A2 was expressed in BL21(DE3) *Escherichia coli* cells and purified using reversible Ca^2+^-dependent binding to negatively charged phospholipid vesicles followed by size exclusion chromatography [76]. A pET23A vector containing the S100A10 sequence (a gift from Volker Gerke) was expressed in BL21(DE3) cells as previously described [77]. Both proteins were stored at 4°C in 20 mM HEPES buffer at pH 7.4, containing 100 mM NaCl with the addition of 1 mM dithiothreitol. Concentrations of all proteins and peptides, including the A2t complex, were determined using bicinchoninic acid assays (Pierce Thermo Scientific, Rockford, IL) compared to measured absorbance of albumin standards at 562 nm. Purified annexin A2 and S100A10 were combined at a molar ratio of 1∶1 and concentrated to over 8 µg/µl. The mixture was incubated overnight at 4°C, and then loaded onto a superdex 200 10/300 GL (GE Healthcare USA) column equilibrated in the previously mentioned HEPES buffer. The eluted A2t peak was collected and subjected to peptide binding assays.

Peptides were purchased and synthesized by Synthetic Biomolecules (San Diego, CA) or Biomer Technology (San Francisco, CA) and HPLC purified to >95% purity.

### Pseudovirions and Virus-Like Particles

Wildtype HPV16 pseudovirions containing GFP reporter were produced by cotransfection of 293TT cells with plasmids encoding codon-optimized HPV16 L1 and L2 and a GFP reporter plasmid (pCIneoGFP) following published procedures [78]. The infectious titer of PsV preparations (in infectious units/mL) was determined by flow cytometric analysis of 293TT cells treated with varying doses of PsV vector stock. Neutralization of PsV were validated in infection assays via pre-incubation with H16.V5 or H16.E70 prior to cellular exposure, and minimal infection rates of less than 1% were observed. To produce pseudovirions with a mutated L2 (108–126) region [28], site-directed mutagenesis was performed using overlapping mutated primers on the bicistronic packaging HPV16 pseudovirion plasmid p16sheLL as a template [78]. Forward primer 5′-AGCGACCCCAGCATCGTGAGCGGTGGTGATGATACCAGCTTCATCGACGCCGGCGCCCCC-3′ and reverse primer 5′-GGGGGCGCCGGCGTCGATGAAGCTGGTATCATCACCACCGCTCACGATGCTGGGGTCGCT-3′ encoding the amino acid substitution of GGDD for LVEE in the L2 capsid region aa 108–111 were used with the QuikChange II XL Site-Directed Mutagenesis Kit (Stratagene, Cedar Creek, TX) according to manufacturer’s instructions. The entire L1 and L2 ORFs of the resulting plasmid were sequenced to confirm mutagenesis of the 108–111 aa L2 region and that no other unexpected mutations were introduced. HPV16 L1–L2(GGDD) PsV containing GFP reporter plasmid were produced as described above for wildtype HPV16 PsV. Confirmation and quantitation of reporter plasmid DNA encapsidation was performed using quantitative real-time PCR following plasmid DNA isolation from pseudovirion preparations by phenol chloroform extraction and ethanol precipitation. L1 content was quantitated by Coomassie Blue staining next to BSA standards following SDS-PAGE. The infectious titer of L2 mutant PsV were determined on 293TT cells as described above for WT PsV.

HPV16L1 VLP and HPV16L1L2 VLP were produced using a recombinant baculovirus expression system in insect cells as previously described [79]. Western blot analyses confirmed the presence of L1 and either the presence or absence of L2, while a neutralizing antibody ELISA and transmission electron microscopy confirmed the presence of intact particles. Coomassie Blue staining following SDS-PAGE was performed to determine protein purity and standardize the concentration of L1 content of the VLP preparations. VLP were validated in internalization assays via pre-incubation with H16.V5 neutralizing antibody prior to cellular exposure, and minimal internalized virus of less than 5% was observed.

### HPV16 PsV Infection Assays

HaCaT cells seeded at 3×10^4^ cells/well or HeLa cells seeded at 2×10^4^ cells/well were incubated overnight in 24-well plates at 37°C. The cells were subsequently incubated with wildtype HPV16 PsV containing a pCIneo-GFP vector at an MOI 100 for HaCaT or MOI of 1 for HeLa. The MOIs were chosen for each cell type that achieved 15–25% GFP-positive cells. Infectivity was scored 48 h post infection by enumerating GFP+ cells by flow cytometry. Cells treated with PsV alone were set to 100% infection and all other treatments were normalized to this value unless otherwise noted. For SLPI blocking experiments, the cells were incubated with increasing amounts of rhu SLPI or BSA (Bio-Rad, Hercules, CA) in PBS for 1 h at 4°C prior to addition of PsV. For antibody blocking experiments, cells were incubated with increasing amounts of an anti- annexin A2 Ab or isotype control (mouse IgG1) for 1 h at 4°C prior to addition of PsV. In each experiment, PsV were mixed with H16.V5 or H16.E70 neutralizing antibody (1/1000 dilution) as a positive control. Infection assays with HPV16 L1–L2(GGDD) mutant PsV containing pCIneoGFP reporter plasmid were carried out on HaCaT cells using an equivalent amount of L1 content as wildtype PsV and an MOI of 100. Data are representative of three or four replicate wells from at least two independent experiments.

### Immunocytochemical Staining and Fluorescence Microscopy

HaCaT and HeLa cells were seeded at 1×10^4^ cells/well on 8 well permanox chamber slides (Thermo Scientific) and incubated at 37°C overnight. Cells were washed and blocked with PBST (0.1% tween 20) containing 5% goat and donkey serum at room temperature (RT). Cells were then incubated with an S100A10 antibody, washed extensively, fixed with 2% paraformaldehyde, and after additional washes were incubated with fluorophore-conjugated secondary antibodies. Lastly, coverslips were applied using Vecta Shield hard mounting media with DAPI (Vector Labs, Bermingham, CA). For control staining, cells were either stained with a mouse or rabbit IgG isotype control (Abcam) followed by fluorophore staining, or the fluorophore-conjugated secondary antibody was used alone. In both cases there was minimal to no fluorescence observed. Images were acquired using an Axio Imager upright confocal microscope using the Axio Imager Bio II software (Zeiss).

### Co-Immunoprecipitation

HeLa cells were grown in 175 cm^2^ culture flasks to 80% confluency (approx. 20×10^6^ cells), then incubated with 125 µg HPV16 PsV, HPV16 L1L2 VLP, or HPV16 L1 VLP in 10 mL PBS (approx. 1.9×10^5^ particles/cell) for 1 hour at 37°C or left untreated. The cells were washed, collected with a cell scraper and spun down at 800 g at 4°C. The cells were then re-suspended with extracellular cross-linking agent DTSSP (3,3′-dithiobis{sulfosuccinimidylproprionate}) (Thermo Scientific) at a concentration of 1.5 mM in PBS for 2 hours at 4°C with rotation. Cells were then washed, and re-suspended in an IP compatible lysis buffer (25 mM Tris-HCl pH 7.4, 150 mM NaCl, 1% NP-40, 1 mM EDTA, 5% Glycerol). HPV16 VLP and PsV were precipitated out of solution with H16.V5 antibody conjugated to magnetic Protein-G Dynabeads (Life Technologies). The precipitated proteins were eluted off Ab-bead complexes under denaturing conditions to monomer form, and the co-immunoprecipitation of annexin A2, S100A10, and L1 protein were analyzed via Western blot. L1 protein was detected with a rabbit polyclonal antibody to prevent cross reactivity with the mouse H16.V5 antibody used to immunoprecipitate the capsids.

### Western Blotting

All samples were electrophoresed on NuPage Novex Bis-Tris gels (Life Technologies), transferred to nitrocellulose membranes and blocked with StartingBlock blocking buffer (Thermo Scientific). Membranes were probed then with anti- annexin A2, S100A10, HPV16 L1 (Camvir-1), HPV16 L2 (DK44214) or GAPDH antibodies. Blots were subsequently incubated with infrared-labeled secondary antibodies. Blots were imaged and bands quantified using the Licor Odyssey Infrared imaging system. For detection of extracellular A2t with EDTA treatment, HaCaT and HeLa cells were grown to 80% confluency in 12-well plates in normal growth media. Cells were washed with PBS followed by incubation with PBS supplemented with Ca^2+^ or PBS with increasing concentration of Ca^2+^ chelating agent EDTA for 45 min to release calcium dependent membrane bound proteins. The supernatants were collected and the presence of A2t was analyzed via immunostaining against annexin A2 and S100A10. For shRNA knockdown experiments, cellular extracts were prepared using Mammalian Protein Extraction Reagent (Pierce) containing Halt Protease Inhibitor Cocktail (Thermo Scientific). Samples were normalized prior to immunostaining via a Bradford protein assay (BioRad) and GAPDH served as a loading control.

### ELISA Assays

For assays to determine accessibility of *L2_108–120_* epitope on capsid surface, 96-well Microlon ELISA plates (USA Scientific, Ocala, FL) were incubated with 500 ng of HPV16 L1 VLP, HPV16 L1L2 VLP or HPV16 PsV in 100 µL PBS overnight at 4°C. The plate was washed with PBST (X% Tween-20) and blocked with 200 µL of PBS containing 3% BSA for 2 h at RT. The plate was washed with PBST and incubated with 1∶1000 16L2.4B4 (anti-L2_108–120_) or 1∶5000 H16.V5 (anti-L1) in PBST with 1% BSA for 2 h at RT. The plate was washed and incubated with 1∶5000 rabbit-anti-mouse HRP in PBST with 1% BSA for 1 h at RT. The plate was washed, and 100 µL of the HRP substrate (o-phenylenediamine) was added. The absorbance was measured at 490 nm with a Hidex Chameleon plate reader. In control experiments, no VLP or PsV were added, and stained with primary, secondary, or both antibodies and minimal absorbance was seen. For assays to determine in vitro binding capacity of HPV16 capsids to purified A2t protein, 500 ng of purified A2t was coated separately on ELISA plates overnight at 4°C. Plates were washed and blocked with 10% casein blocking buffer (Thermo Scientific) for 2 h at RT. Plates were washed, then incubated with 400 ng of WT HPV16 PsV, HPV16 L1–L2(GGDD) mutant PsV, no PsV, or rhu SLPI as a positive control for A2t binding. Bound PsV were detected with conformational anti-L1 H16.V5 antibody, followed by anti-mouse IgG HRP antibody conjugate. Bound SLPI was detected with goat anti-SLPI antibody, followed by anti-goat IgG HRP antibody conjugate and addition of substrate.

### Electron Paramagnetic Resonance

The L2_108–126_ peptides used in our EPR studies were synthesized with a cysteine at the N-terminus, and followed by a spacer of 3 physiological amino acids (C-IVS) preceding the canonical L2_108–126_ and L2 scrambled sequences (C-IVS-LVEETSFIDAGAPTSVPSI and C-IVS-IESPVSDTALGTPEIFVSA respectively). Peptides were combined with 8× molar excess paramagnetic label (1-oxyl-2, 2, 5, 5-tetramethyl-Δ3-pyrroline-3-methyl) (R1) methanethiosulfonate (MTSL), (Toronto Research Chemicals, Canada) and left to react overnight at 4°C. Free label was removed by gel filtration (PD10 GE, United Kingdom). All peptides were solubilized into HEPES buffer. For competition assays, the scrambled peptide was non-paramagnetic labeled with an N-acetylated MTSL paramagnetic-label analog (1-acetyl-2,2,5,5- tetramethyl-Δ3-pyrroline-3-methyl) (R1′) at an 8× molar excess to emulate the paramagnetic label on control peptides. Binding interactions were examined by combining paramagnetically-labeled L2 peptide with A2t, BSA, ANXA2, or S100A10 (20 µM, 100 µM, 100 µM, 100 µM, and 100 µM respectively) in 10 µL volumes of the HEPES buffer, confined by glass capillaries, and measured at room temperature over a period of 24 hours using a Bruker EMX X-band EPR spectrometer. For the cysteine blocked S100A10, S100A10 was treated with R1′ and excess label was removed via gel filtration (see above) before the addition of paramagnetic labeled peptides. The experiments were done in triplicate with measurements taken at 12 db, in 5 scan intervals. All spectra were normalized to the same number of scans. To quantify the amount of bound peptide spectra of all tested proteins in combination with the L2_108–126_ or ScrL2 peptides were compared to the spectra of L2_108–126_ or ScrL2 peptides alone in solution using a spectra analyzing software (EPR 130, University of California, Los Angeles). By subtracting the alone peptide spectra from that of the combined samples we derive the spectra of the bound spectra, and based on double integration, relate this spectra to a concentration of the peptide that bound to the target protein.

### VLP Internalization Assays

HPV16 L1 VLP and HPV16 L1L2 VLP were labeled with CFDA-SE using Vybrant CFDA-SE cell tracer kit (Life Technologies) as directed by the manufacturer’s instructions. After labeling the HPV16 VLP were column filtered with 2% agarose beads size standard 50–150 µm (Agarose Bead Technologies, Tampa, FL) to remove excess free label. HeLa cells were seeded at a concentration of 2×10^5^ cells/well in 12 well plates and incubated at 37°C overnight. Next, CFDA-SE labeled HPV16 L1L2 VLP or HPV16 L1 VLP were incubated with the cells at 37°C for 3 h at a concentration of 1 µg/10^6^ cells (approx. 3×10^4^ particles/cell). The cells were then analyzed via flow cytometry, and the mean fluorescent intensity (MFI) of the CFDA-SE signals was recorded. In control experiments, HeLa cells were incubated with virus neutralizing antibodies H16.V5 or H16.E70 (1∶1000) pre-treated CFDA-SE labeled VLP to ensure VLP integrity and lack of residual free CFDA-SE label, and minimal CFDA-SE signals were observed (<10%). Following 3 h incubation, the cells were washed with PBS and harvested with trypsin-EDTA, washed, fixed with 2% paraformaldehyde and analyzed by flow cytometry. For SLPI blocking experiments, cells were either left untreated or incubated with increasing concentrations of rhu-SLPI in 0.5 mL PBS for 30 min at 4°C prior to addition of CFDA-SE labeled VLPs. Data were normalized to untreated groups.

### Quantitative RT-PCR

Total RNA was isolated from HeLa cell populations using an RNeasy Mini kit (Qiagen) according to the manufacturer’s instructions. The iScript cDNA Synthesis Kit (Bio-Rad) was used for reverse transcription of total isolated RNA to cDNA. An iQ SYBR Green Supermix (Bio-Rad) was used for quantitative real-time PCR with the following primer sequences: ANXA2, 5′-TCGGACACATCTGGTGACTTCC-3′ (sense), 5′-CCTCTTCACTCCAGCGTCATAG-3′ (antisense); S100A10, 5′-AACAAAGGAGGACCTGAGAGTAC-3′ (sense), 5′-CTTTGCCATCTCTACACTGGTCC-3′ (antisense); GAPDH, 5′-TGGGCTACACTGAGCACCAG-3′ (sense), 5′-GAGGAGGTGGAAACTGCGAC-3′ (antisense). Samples were run on a CFX96 real-time PCR system (Bio-Rad) operated by CFX Manager software (version 1.5). Melt curve analysis for each primer pair was performed after cycling and data capture to ensure primer specificity. Relative ANXA2 and S100A10 gene expression was analyzed with CFX Manager software with normalization to the GAPDH reference gene (ΔΔCq). For real-time PCR measurement of encapsidated GFP reporter DNA from PsV preps, plasmid DNA containing the GFP reporter was purified from 50 µL of WT or mutant PsV preps using phenol chloroform extraction and ethanol precipitation. Reporter plasmid DNA was also isolated using the QIAamp MinElute Virus Spin Kit (Qiagen) with similar results. Real-time PCR reactions were performed in triplicate using 5 µL of extracted DNA template with iQ SYBR Green Supermix and 500 nM each of forward (5′-AACTACAACGCCCACAATGTGT-3′) and reverse (5′-CGGATCTTGAAGTTCACCTTGAT-3′) primers for GFP. Quantitation was performed against a standard curve generated with purified pCIneoGFP plasmid template ranging from 10^7^ copies to 10^3^ copies per reaction.

### shRNA-mediated Down Regulation of A2t

HeLa cells were transduced at MOI 1 with doxycycline-inducible pTRIPZ Lentiviral construct encoding a Human anti-ANXA2 shRNA (OpenBiosystems; mature sense GCAAGTCCCTGTACTATTA/mature antisense TAATAGTACAGGGACTTGC) or a non-silencing pTRIPZ Tet-On analog Lentiviral control following the manufacturer’s protocol. Both pTRIPZ vectors express red fluorescent protein when induced, allowing for visual confirmation of transduction. Stably transduced cells were selected by using puromycin. Clones were expanded from a single transduced cell by limiting dilution and either treated with doxycycline (1 µg/mL) for a minimum of one week to induce shRNA expression and ANXA2 knockdown or left untreated to control for vector integration effects.

### Statistical Analysis

All statistical analyses were performed using GraphPad Prism (GraphPad Software Inc., San Diego, CA).

## Supporting Information

Figure S1 **HPV16 L1 and L2 immunoblot analysis of HPV16 WT PsV and HPV16 L1–L2(GGDD) mutant PsV.** An equal amount of infectious particles of wildtype and mutant pseudovirions were separated by SDS-PAGE and transferred to PVDF membranes. Blots were separately probed with a mouse anti-L1 antibody followed by IR800 (green)-labeled goat anti-mouse IgG secondary antibody, or a rabbit anti-HPV16 L2 antibody followed by AlexaFluor 680 (red)-labeled goat anti-rabbit IgG secondary antibody. Blots were scanned on the Licor Odyssey infrared imaging system. The L2 immunoblot shows that incorporation of the L2 protein into pseudovirions is not affected by the L2_108–111_ LVEE → GGDD mutation.
(TIFF)
