# Supplementary figures and images for: The S100A10 Subunit of the Annexin A2 Heterotetramer Facilitates L2-Mediated Human Papillomavirus Infection
Source: PLoS One. 2012 Aug 22;7(8):e43519. doi: 10.1371/journal.pone.0043519 (PMC3425544; doi:10.1371/journal.pone.0043519)

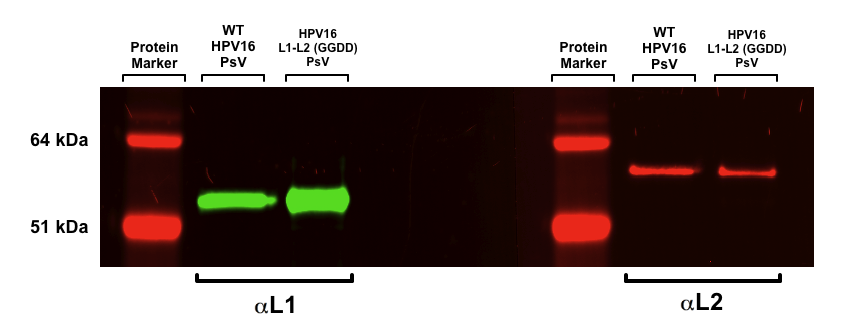

Supplement: Figure S1 — HPV16 L1 and L2 immunoblot analysis of HPV16 WT PsV and HPV16 L1–L2(GGDD) mutant PsV. An equal amount of infectious particles of wildtype and mutant pseudovirions were separated by SDS-PAGE and transferred to PVDF membranes. Blots were separately probed with a mouse anti-L1 antibody followed by IR800 (green)-labeled goat anti-mouse IgG secondary antibody, or a rabbit anti-HPV16 L2 antibody followed by AlexaFluor 680 (red)-labeled goat anti-rabbit IgG secondary antibody. Blots were scanned on the Licor Odyssey infrared imaging system. The L2 immunoblot shows that incorporation of the L2 protein into pseudovirions is not affected by the L2108–111 LVEE → GGDD mutation. (TIFF) [file pone.0043519.s001.tiff]
